# Clarification of Findings in Key Points

**DOI:** 10.1001/jamanetworkopen.2026.17937

**Published:** 2026-05-01

In the Original Investigation “Sequential Safety Surveillance of RSVpreF Vaccination During Pregnancy Early in the Postapproval Period,”^1^ published in *JAMA Network Open* on April 21, 2026, clarifications to the findings of this study were made to the Key Points.

## References

[1] Michnick AI, MacDonald SC, Adimadhyam S, . Sequential Safety Surveillance of RSVpreF Vaccination During Pregnancy Early in the Postapproval Period. JAMA Netw Open. 2026;9(4):e266190. doi:10.1001/jamanetworkopen.2026.6190 42012833 PMC13100873

